# Where Do Cancer Patients in Receipt of Home-Based Palliative Care Prefer to Die and What Are the Determinants of a Preference for a Home Death?

**DOI:** 10.3390/ijerph18010235

**Published:** 2020-12-30

**Authors:** Jiaoli Cai, Li Zhang, Denise Guerriere, Hongli Fan, Peter C. Coyte

**Affiliations:** 1 School of Economics and Management, Beijing Jiaotong University, No. 3 Shangyuancun, Haidian District, Beijing 100044, China; jiaoli.cai@bjtu.edu.cn (J.C.); lzhang@bjtu.edu.cn (L.Z.); 2 Institute of Health Policy, Management and Evaluation, University of Toronto, Health Sciences Building, 155 College Street, Suite 425, Toronto, ON M5T 3M6, Canada; denise.guerriere@utoronto.ca (D.G.); peter.coyte@utoronto.ca (P.C.C.); 3 School of Insurance, Shandong University of Finance and Economics, No. 40 Shungeng Road, Shizhong District, Jinan 250000, China

**Keywords:** cancer, home-based care, palliative care, preference for place of death, home care services

## Abstract

Understanding the preferred place of death may assist to organize and deliver palliative health care services. The study aims to assess preference for place of death among cancer patients in receipt of home-based palliative care, and to determine the variables that affect their preference for a home death. A prospective cohort design was carried out from July 2010 to August 2012. Over the course of their palliative care trajectory, a total of 303 family caregivers of cancer patients were interviewed. Multivariate regression analysis was employed to assess the determinants of a preferred home death. The majority (65%) of patients had a preference of home death. The intensity of home-based physician visits and home-based personal support worker (PSW) care promotes a preference for a home death. Married patients, patients receiving post-graduate education and patients with higher Palliative Performance Scale (PPS) scores were more likely to have a preference of home death. Patients reduced the likelihood of preferring a home death when their family caregiver had high burden. This study suggests that the majority of cancer patients have a preference of home death. Health mangers and policy makers have the potential to develop policies that facilitate those preferences.

## 1. Introduction

Following health system restructuring in Canada, more attention has been directed towards home-based palliative care. In Ontario, palliative home services are currently publicly funded services. Home-based palliative care programs provide community and team-based multidisciplinary care to care recipients at home. Care components primarily comprise home-based physician visits, nursing visits and hours of care from personal support workers (PSW) [1]. Home-based palliative care enables patients who are terminally ill to be cared for at home and aims to improve their quality of life [2]. Many people prefer to be taken care of at home and also prefer a death at home compared to other settings, such as a hospital or hospice [3,4]. A better understanding of the determinants of preference for place of death may assist to actualize those preferences, while achieving a preferred place of death is considered an indicator of high-quality end-of-life care [5,6]. Recognizing individual preference for the place of death can improve the quality of end-of-life care and help to provide support for them [7].

Patients’ preferences for the place of death have been examined in various countries or regions, such as China [8], Egypt [9], Netherlands [10], Denmark [4,11], Japan [7], UK [3,12], Canada [13,14], and Taiwan [15] and these studies indicate that most patients preferred to die at home. Some of these studies have identified the determinants of preferred place of death [7,8,9,11,15]. Multiple factors such as patient’s [11] or caregiver’s [9] demographic characteristics, cancer type [15], patient’s functional status [7,8,9,15], socio-economic factors [11] and living arrangements [8] have been reported to affect patient’s preferred place of death. For example, Gu et al. conducted a multivariable logistic regression analysis and found that cancer patients living in rural areas in China, living with their relatives, and of lower educational attainment were more likely to prefer to die at home [8]. A study conducted in Egypt found that cancer patients with poorer performance status and cared for by fully employed caregivers were related to preferring to die in hospital [9]. A study conducted in Taiwan found that cancer patients preferred to die at home when they were aware of their prognosis, had greater functional dependency, had a lower level of education, had either liver/pancreatic or head/neck cancer, or their family members knew their preferred place of death [15]. Another study conducted in Denmark found a significant positive association between being male and preference for a home death, but a negative relationship between having medium income (VS. high income) and preference for a home death [11]. However, these studies neither focused on home-based palliative care [7,8,15], nor specifically emphasized a preferred home death [9]. Little is known about the variables that affected home-based cancer patients’ preferences for a home death in Canada. The study aimed to assess preference for place of death among cancer patients who received home-based palliative care in Ontario, Canada, and to determine the variables that affect patients’ preference for a home death.

## 2. Methods

### 2.1. Study Design

A prospective cohort design was used to determine the variables related to a preferred home death among cancer patients in two home-based palliative care programs in urban locations in Ontario, Canada. The two programs were the Temmy Latner for Palliative Care at Mount Sinai Hospital (Toronto); and the Hamilton Niagara Haldimand Brant Palliative Care Teams. They both are in large ethnically diverse urban communities. The team consists of program-dedicated palliative care physicians, home care agencies such as nursing services, outpatient clinics, family practice physicians, etc. [16]. These programs provide integrated community-based and team-based multidisciplinary palliative care 24 h/day, 7 days/week to those patients who were at home. Caregiver participants were chosen to participate in the study if they had the three characteristics. They were at least 18 years old, primary caregivers of patients, and spoke fluent English. Those who verbally agreed to participate filled out a written consent form.

During the study period, from July 2010 to August 2012, data were collected from caregiver participants using telephone interviews. From entering the palliative care program to the death of the patient, participants received an interview every two weeks. We selected a two-week period to minimize recall bias and also to avoid overburdening caregivers [16]. The caregiver participants were asked to report health service utilization, patient’s functional status and caregiver burden over the previous two weeks. This study was approved by the Ethical Review Boards of University of Toronto and Mount Sinai Hospital which is in line with the ethical principles embedded in the Declaration of Helsinki [17].

### 2.2. Measures

The selection of potential determinants of a preferred home death was guided by a systematic review of the literature concerning the variables related to a preferred place of death [18] and a review of variables associated with a home death [19]. The potential determinants were divided into three groups, comprising environmental, illness-related, and individual factors. The dependent variable was a preferred home death. The data on a patient’s preferred place of death were collected by the patient’s attending palliative care physician in the patient’s home during their first home meeting with the patient. So, only the baseline preference for place of death was reported by patients and this preference was used in the study. The independent variables included the socio-demographic characteristics of patients and caregivers and health services utilization. Patient information, such as sex, age, marital status, living arrangements, comorbidity score, level of education, was collected using the unique health card number of the patient. Caregiver information, such as their sex, age, level of education, and the type of caregivers (spouse or others) was recorded in the first interview. While health service utilization, patient’s functional status and caregiver burden were recorded at each interview over the palliative care trajectory, only the data from the first interview (the baseline data) were used in the descriptive and regression analyses.

The Ambulatory and Home Care Record (AHCR) (© Coyte and Guerriere 1998) was employed to collect health services utilization at each interview. The AHCR recorded the use of health services which mainly included home-based physician and nursing visits, home-based hours of PSW care, and emergency department visits [1]. Caregivers recalled the propensity and intensity of each service utilization over the prior 2 weeks. The AHCR has been validated and applied in various clinical settings [20]. There is a moderate to high agreement between the responses of participants and the administrative data, with a kappa value from 0.41 to 1.00 [20]. While data on home-based physician and nursing visits, home-based hours of PSW care, and emergency department visits were collected at each interview, in this study we only used the baseline data in all of our analyses including the descriptive and regression analyses.

Patient’s functional status was measured by the Palliative Performance Scale (PPS) score at each interview. The PPS score ranges from 0 to 100, and a higher value means a better functional status. The intra-class correlation coefficients were calculated based on the reliability test and between 0.93 to 0.96 [21,22].

The Caregiver Burden Scale in the End-of-Life Care (CBS-EOLC) was a measure of caregiving burden of caregivers [23]. The CBS-EOLC is a 4-point Likert scale and gets a score between 16 to 64. A high value represents a high burden. There was an appropriate reliability (Cronbach’s alpha = 0.95), good levels of convergent validity with fatigue (r = 0.69) and depression (r = 0.54) based on the evaluation of the psychometric properties of the scale [23]. 

### 2.3. Data Analysis

Descriptive statistics were used to show the characteristics of the patients and caregivers. Cross-tabulation and a Pearson chi-square test were used to show differences between the preferences for death at home and the preference for death outside the home. In the regression analysis, the outcome variable was the preference for a home death. Multivariate analysis with a backward stepwise regression was used to examine variables associated with the outcome variable, while taking into account multiple variables. The multivariate analysis model included all variables with a significant level of 10%. Logistic regression was employed. Stata 13.0 for Mac (StataCorp LP, College Station, TX, USA) deals with all analyses.

## 3. Results

**Figure 1:**
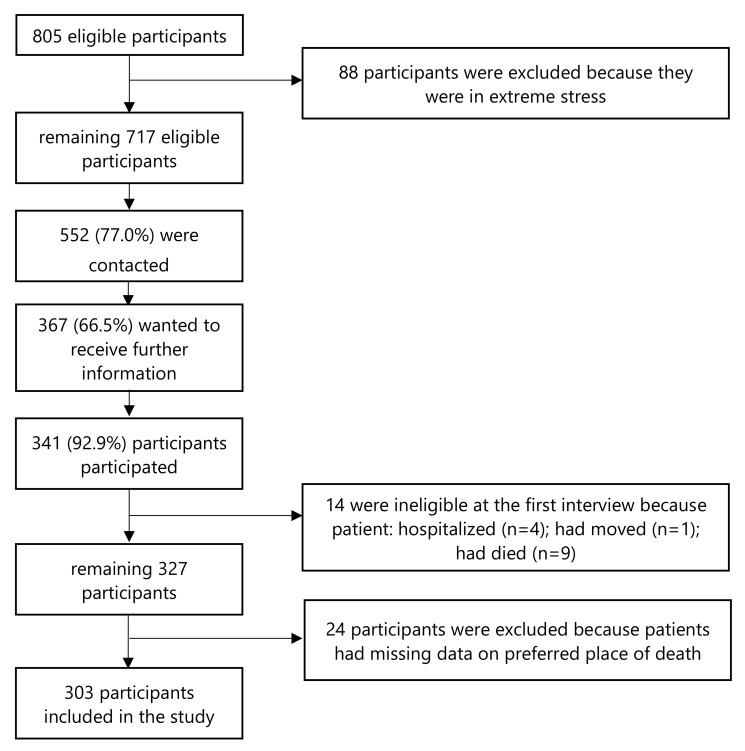
Diagram of inclusion/exclusion criteria of the study.

During the study period, from July 2010 to August 2012, a total of 805 eligible caregiver-participants were identified for the study. Since the attending physicians noticed that 88 participants were under stress, these participants were considered inappropriate for the study. 552 (77.0%) of the remaining 717 participants were contacted; 367 (66.5%) would like to know more about the study, and finally, 341 (92.9%) potential participants agreed to participate after receiving the study information. However, because 4 patients was hospitalized, 1 patient had moved and 9 patients had died at the first interview, 14 ineligible participants were excluded [24]. An additional 24 participants were excluded because pertinent data were missing on patients’ preferred place of death. Consequently, the sample for this study consists of 303 caregiver-participants (Figure 1). From the date patients entered the palliative care programs to their death, the average survival time of the patients was 109 days. 

Table 1 presents patients’ and caregivers’ characteristics and the distribution of patients’ preference for place of death. Among the 303 patients, 197 (65.0%) patients had a preference of home death. The mean age of patients and caregivers were 72.4 (SD: 12.38) and 59.5 (SD: 12.99). The baseline PPS score for most patients (67.66%) was below 30, indicating that most patients were in poor functional status when they entered the palliative care program. A significant difference exists in the patient’s marital status, level of education, PPS score and the caregiver’s age and level of education between patients who preferred a home death and those preferred a death outside the home. The patients with a preference of home death were more likely to be those who received home-based physician visits and hours of PSW care.

Table 2 shows the multivariate regression results. Six variables were predictors of preferring a home death. Compared to married patients, those who were divorced, separated, widowed (OR 0.56; 95% CI 0.32, 0.98), or never married (OR 0.18; 95% CI 0.04, 0.81) were less likely to report a preference of home death. Compared to the patients who received high school education or less, those who received post-graduate education (OR 3.64; 95% CI 1.01, 13.07) were more likely to report a preference for a home death. Patients with higher PPS scores were more likely to prefer a home death (OR 1.08; 95% CI 1.03, 1.13). The higher the burden on the caregiver, the less likely the patient was to prefer a death at home (OR 0.96; 95% CI 0.93, 1.00). An increase in the intensity of home-based physician visits (OR 1.49, 95% CI 1.03, 2.16) and hours of home-based PSW care (OR 1.20; 95% CI 0.97, 1.50) increased the likelihood that patients would report a preferred home death. 

## 4. Discussion

### 4.1. Main Findings and Implications for Clinical Practice

This study found that 65% of patients preferred to die at home. This majority may be due to home-based palliative care providing formal care for patients at home, thus facilitating patients to be taken care of and to die at home. Additionally, perhaps patients find physical and emotional comfort at home [25]. The results are similar to previous studies which also reported that most patients preferred a home death [3,9,12,26].

Our results showed that an increase in the intensity of home-based palliative physician visits and PSW hours increased the likelihood of patients reporting a preference for a home death. This finding may be that the use of home-based palliative care services may increase the satisfaction of patients and reduce caregiver burden. Previous studies reported that home-based palliative care programs helped to avoid hospital use and reduced the total healthcare costs [27], increased the quality of life of end-stage heart failure (ESHF) patients and patient’s satisfaction with care, but reduced caregiver burden [2]. An enhanced intensity of formal care provided by home-based palliative physicians and PSWs may also better control patients’ pain and other symptoms thereby leading to a preference for settings offering such intensity of care. Previous studies have found that the availability of a palliative home care team [28] and high PSW costs [29] help patients to die at home. This might explain why patients preferred to die at home after receiving more support from home-based palliative physician visits and hours of PSW services. We did not identify that the previous studies had explored the impact of the intensity of home-based palliative physician visits and hours of PSW care, making comparisons with previous work impossible.

We found that patients were more likely to report a preference of home death when they had higher PPS scores. This might be because higher PPS scores indicate better health and a greater chance that home care can meet these patients’ needs. Another possible explanation is that patients with poor functional status were worried about causing heavy care burden to informal caregivers, thus not preferring a home death. Our results echoes a Japanese study [7], which showed that good self-rated health was related to a preferred home death among elderly people. However, our study cannot be directly compared to the Japanese study because that study was aimed at only the elderly. Our results contrast with Gu et al.’s study in China and Chen et al.’s study in Taiwan, both of which found that terminally ill patients in poorer functional health (measure by Karnofsky Performance Status and Enforced Social Dependency Scale score, respectively) were more likely to prefer a home death [8,15]. Gu et al. argued that perhaps patients with poorer health lack confidence to alleviate pain and would like to spend time with their family at home in the final stages of life [8].

Compared to married patients, patients who were divorced, separated, widowed or never married decreased the likelihood of preferring a home death. This finding may be due to the absence of a partner to provide informal care as well as to offer emotional support. One previous study conducted in the U.S. assessed 458 adult patients receiving the general medical service and found similar results to our study [30]. However, the American study was focused on hospitalized adults and was not focused on palliative care programs. Our results also differ from the studies conducted in Egypt and Taiwan, which reported that marital status was not related to a preferred place of death [9,15].

We found that patients receiving post-graduate education tended to have a preference of home death. Perhaps patients with a higher level of education have access to home-based care due to their financial resources [8] and are better advocates for their care. A previous study reported that low levels of education were related to reduced access to specialist palliative care services [31]. Our findings contrast with the Chinese studies [8,15] that found that patients with lower levels of education tended to have a preference of home death. Gu et al. reported that in China, patients with lower levels of education may have been more susceptible to traditional Chinese cultural influences, so they think home is the most appropriate place of death [8]. Another previous study found that there was no relationship between cancer patient’s highest educational level and preference of a home death [11].

We found that patients who had caregivers with higher caregiving burden were less likely to report a preference for a home death. The reason for this observation may be that patients may not want their family caregivers to have the continued burden of care, or because family caregivers were unable to meet all of the patients’ caregiving needs at home. A Japanese study reported that the concerns about family burden reduced the likelihood of cancer patients choosing home as their preferred location of care [32].

### 4.2. Limitations of the Study

Several study limitations should be paid attention to. First, the programs did not record whether the preferred place of death changed over the course of the palliative care trajectory. Therefore, we cannot analyze how these preferences may have changed. Previous studies showed that there were divergent results about changes in preference for places of death. A study examined the extent of change in preference for place of death among patients with symptoms of advanced heart failure, and found that 66% of the patients have changed their preference at least once [33]. Another review study showed that the majority of patients’ preference for place of death did not change over time [34]. Neergaard et al. found that the ideal preference for place of death changed significantly over time, while patient’s realistic preferences were more stable [35]. In our study, the patients’ preference for place of death would recognize all the realities associated with the patient’s circumstances and therefore, reflects their “realistic” preferences for the place of death. As such, we believe our results are robust to this consideration. Second, the data of our study were from two home-based palliative programs in Ontario, Canada. We, therefore, caution the reader not to generalize our findings to other palliative care settings. However, generalizability can be improved because of the diverse demographic and ethnic background of those patients in the programs. Third, some data were recalled by caregivers, which may cause a bias in social desirability and recall [36]. However, we believe these potential biases are small because there are acceptable standards of the psychometric properties of the study instruments. Fourth, while the overall study from which our data were derived was longitudinal in design, only the baseline interview data were used in the reported analysis. As such, the current study is more appropriately characterized as cross-sectional in design.

## 5. Conclusions

This prospective cohort study was designed to assess preference for place of death among cancer patients receiving home-based palliative care, and to determine the variables that affect their preference for a home death. The overwhelming majority (65%) of the cancer patients preferred to die at home. This finding suggests that if home is the preferred place of death for cancer patients, health mangers and policy makers have the potential to develop policies that facilitate those preferences. Caregiver burden reduced the likelihood of a patients’ preference for a home death. Suitable interventions may be developed for those caregivers in order to alleviate caregiving burden, thus reducing its impact on patients’ preference for a home death. Policy makers may also need to focus more on patients with lower PPS, as they were less likely to prefer to die at home. Perhaps it was because their care needs were not being met at home. The intensity of home-based palliative physician visits and hours of PSW care were important variables associated with a preferred home death. Efforts should be expended to ensure that patients have timely access to home-based physician and PSW services in order to respect and facilitate a patient’s preference for a home death. Where patients prefer to die and where they prefer to receive care are two different concepts. Further research may address the factors that affect the patient’s preference for location of care. In addition, not all patients prefer to die at home. In the future, research may analyze the influencing factors of preference for other places, such as hospital death. Further research that assesses the variables that promote the actualization of preferred place of death could also be considered. What is more, public and health care providers are encouraged to strengthen their communications with patients and their caregivers in order to understand their needs and to respect their preferences for the place of death.

## Figures and Tables

**Table 1:** Patient and Caregiver Characteristics (*n* = 303).

	Frequency (Percent)			
Variables	All	Preferred Death at Home	Preferred Death Outside Home	*p*-Value
Individual Factors (decedent)	303	197	106	
Age (year): mean 72.4, median 73, SD 12.38				
31–64	85 (28.05)	53 (26.90)	32 (30.19)	0.827
65–79	113 (37.29)	75 (38.07)	38 (35.85)	
80–96	105 (34.65)	69 (35.03)	36 (33.96)	
Sex				
Female	164 (54.13)	103 (52.28)	61 (57.55)	0.381
Male	139 (45.87)	94 (47.72)	45 (42.45)	
Marital status				
Married	178 (58.75)	125 (63.45)	53 (50.00)	0.022
Divorced, separated, widowed	116 (38.28)	69 (35.03)	47 (44.34)	
Never married	9 (2.97)	3 (1.52)	6 (5.66)	
Deprivation score: mean 0.73, median 0.70, SD 0.42				
Quartile 1 (<0.45)	49 (16.17)	35 (17.77)	14 (13.21)	0.703
Quartile 2 (0.45–0.68)	49 (16.17)	33 (16.75)	16 (15.09)	
Quartile 3 (0.70–1.00)	49 (16.17)	37 (18.78)	12 (11.32)	
Quartile 4 (1.04–2.23)	48 (15.84)	37 (18.78)	11 (10.38)	
Missing	108 (35.64)	55 (27.92)	53 (50.00)	
Education level				
High school or less	161 (53.14)	97 (49.24)	64 (60.38)	0.044
Any university	116 (38.28)	78 (39.59)	38 (35.85)	
Post-graduate	26 (8.58)	22 (11.17)	4 (3.77)	
Environmental Factors				
Living arrangement				
Live with others	250 (82.51)	166 (84.26)	84 (79.25)	0.273
Live alone	53 (17.49)	31 (15.74)	22 (20.75)	
Formal care ^1^				
Home-based physician visits: mean 1.21, median 1, SD 0.94				
Yes	246 (81.19)	169 (85.79)	77 (72.64)	0.005
No	57 (18.81)	28 (14.21)	29 (27.36)	
Home-based nurse visits: mean 6.12, median 4, SD 5.99				
Yes	288 (95.05)	189 (95.94)	99 (93.40)	0.330
No	15 (4.95)	8 (4.06)	7 (6.60)	
Home-based PSW hours: mean 1.09, median 0.28, SD 1.70				
Yes	156 (51.49)	112 (56.85)	44 (41.51)	0.011
No	147 (48.51)	85 (43.15)	62 (58.49)	
Emergency visits: mean 0.16, median 0, SD 0.56				
Yes	33 (10.89)	23 (11.68)	10 (9.43)	0.550
No	270 (89.11)	174 (88.32)	96 (90.57)	
Caregiver characteristics				
Age (year): mean 59.5, median 60.5, SD 12.99				
20–45	39 (12.87)	24 (12.18)	15 (14.15)	0.063
46–64	159 (52.48)	96 (48.73)	63 (59.43)	
65–94	104 (34.32)	77 (39.09)	27 (25.47)	
Missing	1 (0.33)	0 (0.00)	1 (0.94)	
Sex				
Female	206 (67.99)	137 (69.54)	69 (65.09)	0.429
Male	97 (32.01)	60 (30.46)	37 (34.91)	
Relationship to patient				
Spouse	146 (48.18)	101 (51.27)	45 (42.45)	0.164
Others	156 (51.49)	96 (48.73)	60 (56.60)	
Missing	1 (0.33)	0 (0.00)	1 (0.94)	
Employment status				
Retired	110 (36.30)	69 (35.03)	41 (38.68)	0.528
Others	193 (63.70)	128 (64.97)	65 (61.32)	
Education level				
High school or less	161 (53.14)	97 (49.24)	64 (60.38)	0.043
Any university	52 (17.16)	32 (16.24)	20 (18.87)	
Post-graduate	90 (29.70)	68 (34.52)	22 (20.75)	
Caregiver burden ^1^: mean 26.01, median 24.5, SD 7.56				
16–20	73 (24.09)	45 (22.84)	28 (26.42)	0.960
21–24	69 (22.77)	46 (23.35)	23 (21.70)	
25–30	76 (25.08)	51 (25.89)	25 (23.58)	
31–57	66 (21.78)	43 (21.83)	23 (21.70)	
Missing	19 (6.27)	12 (6.09)	7 (6.60)	
Illness-related Factors				
Comorbidity score: mean 6.72, median 6, SD 1.02				
6–7	250 (82.51)	160 (81.22)	90 (84.91)	0.637
8–9	48 (15.84)	33 (16.75)	15 (14.15)	
10–12	5 (1.65)	4 (2.03)	1 (0.94)	
Baseline PPS ^1^: mean 27.09, median 27, SD 7.62				
8–30	205 (67.66)	121 (61.42)	84 (79.25)	0.002
31–50	98 (32.34)	76 (38.58)	22 (20.75)	

SD: Standard deviation; PSW: Personal support worker; PPS: Palliative Performance Scale. ^1^ The value at first interview.

**Table 2:** Multivariate odds ratios for likelihood of preferred home death.

Variables	OR	LCI	UCI	*p*-Value
Individual Factors (decedent)				
Marital status				
Married	Ref			
Divorced, separated, widowed	0.56	0.32	0.98	0.042
Never married	0.18	0.04	0.81	0.026
Education level				
High school or less	Ref			
Any university	1.20	0.68	2.12	0.532
Post-graduate	3.64	1.01	13.07	0.048
Environmental Factors				
Formal care ^1^				
Home-based physician visits (I)	1.49	1.03	2.16	0.035
Home-based PSW hours (I)	1.20	0.97	1.50	0.098
Caregiver characteristics				
Caregiver burden ^1^	0.96	0.93	1.00	0.043
Illness-related Factors				
Baseline PPS ^1^	1.08	1.03	1.13	0.001

OR: odds ratio; LCI, lower confidence intervals; UCI, upper confidence intervals; PSW: personal support worker; PPS: Palliative Performance Scale. I: Intensity, the number of services that patient used. ^1^ The value at first interview.

## Data Availability

The data presented in this study are available on request from the corresponding author. The data are not publicly available due to privacy or ethical restrictions.
